# A study of the factors which influence digital transformation in Kibs companies

**DOI:** 10.3389/fpsyg.2022.993972

**Published:** 2022-12-20

**Authors:** Jorge Alberto Marino-Romero, Pedro Ramiro Palos-Sanchez, Félix Antonio Velicia-Martin, Ricardo Gouveia Rodrigues

**Affiliations:** ^1^Department of Business Administration and Marketing, University of Seville Faculty of Economics and Business Sciences, Seville, Spain; ^2^Department of Financial Economics and Accounting, University of Extremadura, Cáceres, Spain; ^3^Department of Financial Economy and Operation Management, University of Seville Faculty of Economics and Business Sciences, Sevilla, Spain; ^4^NECE-Research Center in Business Sciences, University of Beira Interior, Covilhã, Portugal

**Keywords:** digital transformation, qualitative analysis, grounded theory, professional grouping, KIBS

## Abstract

Eighteen interviews were used in this research to inductively conceptualize the factors that influence digital transformation (DT) in Kibs companies that provide multidisciplinary Knowledge Intensive Business Services. Two main groups were identified: factors of DT and use in the new digital environment. Using the findings obtained, a comparison was made with the existing literature and the most relevant aspects of DT as a disruptive phenomenon which can generate intra-organizational competitive advantage are exposed.

**Objective:**

To identify the factors of innovation-oriented organizational management, generated by the collaboration of the professional grouping of Kibs companies through the systematization of knowledge, which serve to conceptually delimit the DT phenomenon. Ultimately, it is expected to establish recommendations for this type of companies based on providing services with high knowledge value that strive to digitally transform their businesses.

**Originality:**

the paper contributes to advancing the conceptual understanding of DT through the study of Kibs companies, which remain understudied. Likewise, there is no known study that analyzes the factors that give rise to DT in a professional grouping of small Kibs companies. It is clear that this union of small companies generates a strong internal capacity for knowledge absorption, through daily interactions with clients and public administrations, which favors the process of implementing certain technological and strategic components that are beneficial for the development of professional activity and increases the propensity to innovate.

**Methodology:**

qualitative content was analysed using a grounded theory methodology including interviews with experts and the managers of the Kibs companies in the professional sector to obtain a solid basis that can be used to identify the most relevant factors of DT.

**Findings/results:**

as DT is a multidimensional phenomenon of individual companies, this study presents a conceptual framework for the term with the strategic requirements of the market, organizations, public institutions and technological infrastructures of the professional sector. By considering the disruptive factors of digital development in this macroenvironment, conclusions can be made about the basic principles and effects of DT.

## Introduction

Digital transformation (DT) is a phenomenon that tries to provide solutions to the profound changes that originate in society and in the production sector with the use of digital technologies (Majchrzak et al., 2016). Organizations must look for strategies to innovate with these technologies and accept all the implications of DT to obtain optimal operational performance (Hess et al., 2016, p: 123).

Scientific literature has focussed on DT because it is a concept with multiple meanings. Research has basically contributed to understanding different elements of this term, demonstrating that technology itself is only one aspect of the complex network that organizations must manage to be competitive in a digital world (Jia et al., 2018).

Services are a determining factor in developed economies and this has caused an increase in academic interest in this sector. It accounted for 70% of the added value of the sector in Japan, 73.10% in the European Union and 80.40% in the US in 2020 (OECD, 2021). Scientists consider that there is still a large amount to be learnt about service innovation (Frishammar et al., 2012), since technological progress does not only occur in industry but also in the service sector (Brynjolfsson and Mcfee, 2014). It is also affecting knowledge-intensive areas (Susskind, 2017) and, therefore, Kibs companies which develop and provide services by integrating the knowledge gained from different sources (Bolisani et al., 2016). These services are usually developed with intense collaborative relationships between Kibs companies and their customers, which is one of their main characteristics (Chichkanov, 2021). Customers are considered collaborators when producing these services, as they are a very valuable source of external knowledge that enhances innovation (Scarso and Bolisani, 2012). To find the impact of the knowledge gained during the interactions with the Kibs customer, one the most dominant currents in the scientific literature about innovation, artificial intelligence, was studied (Dahlander and Gann, 2010). This approach is motivated by the increase in investment in Information and Communication Technology by organizations which aim to improve productivity and quality in response to customer needs, while trying to reduce operating costs (Scuotto et al., 2017).

Technology is also a relevant element in Kibs companies as studies show that there is a moderate positive relationship between the degree of technological innovation and the level of innovation (Carvalho and Sarkar, 2018). Value is created for customers and companies with different levels of maturity and acceptance in the market (Manyika et al., 2013). The technologies implemented in Kibs companies in the service sector have been grouped into two types in the scientific literature (Brynjolfsson and Mcfee, 2014). The first type includes the improvements produced by machines, including different technology such as artificial intelligence, big data, augmented reality and advanced robotics. The second type focusses on the increase in connectivity with technology such as mobile internet, social networks, Skype, internet of things, cloud computing and fog computing, as well as blockchain (Breunig and Skjølsvik, 2017, p: 4).

To implement technological innovation in Kibs companies in a digitalized environment and meet the demands of a rapidly changing market, organizations need professionals with leadership skills to react optimally to the changes adopted (Summa, 2016). In principle, the faster companies adapt, the more likely they are to gain an advantage over their competitors (Summa, 2016). Leadership and behaviour can be considered as one of the most important prerequisites of an increased capacity of innovation. Leadership is at the heart of promoting organisational innovation in a Kibs organisation.

The research gaps are centered on the divergence of results presented by the studies on the components of DT to be implemented in Kibs companies. This is due to the fact that there is a very heterogeneous set of companies in terms of size, operation and type of activity. Consequently, small service firms find it of little interest to pursue strategies for implementing innovation processes (Vermeulen et al., 2005), and specifically professional service firms tend to consider innovation with a low priority (Brooks et al., 2018). In general, these types of firms prefer to adopt strategic cost-cutting measures for producing immediate results. Moreover, they seem to have limited competence in defining an enabling environment for process innovation and although they maintain close relationships with their customers they seem to have difficulties in translating this commitment into value (Ashok, 2018). Finally, the impact of DT on the absorption of knowledge related to the innovation process has not been investigated.

The DT phenomenon is going to be studied taking into account the macro-environment in which the professional grouping of Kibs and its relationship with governmental Institutions are involved. It is necessary to deepen the conceptualization of DT (Markus and Rowe, 2021) due to the lack of understanding of this concept, which affects multiple organizational levels (companies, markets, public institutions) and its scope requires various levels of analysis (Vial, 2019). In this research DT will be analysed by taking into account the macro-environment of Kibs and its relationship with government institutions. The following research questions were raised: What aspects of DT are incorporated in the Kibs companies of a professional sector? How does the professional group of Kibs companies respond to digital disruption in a competitive environment?

The document has the following structure. In the second section, the parts of DT that affect the business processes of Kibs and that correspond to the changes that occur in their business environment are identified in the theoretical framework. Three elements are taken into consideration: digital technologies, open innovation (OI) oriented business models and leadership. The third section describes the grounded theory methodology used in this research. Sections 4 and 5 present and discuss the most relevant results of the analysis and finally, section 6 explains the conclusions.

## Literature review

The documents were selected using a systematic review of the literature with the aim of identifying the most relevant elements that define DT in the current business environment.

The selection process chose relevant scientific production from the documents contained in the Wos and Scopus databases and used the PRISMA methodology (Liberati et al., 2009) to find the studies with the greatest scientific impact on the business management of SMEs. The different selection stages of the literature review were (Object, context of review and Selection of records by filtering with eligibility criteria).

The following search criteria were used to find the relevant papers for this investigation: 1st, texts published between 2000 and 2021, 2nd, only publications written in the English language, 3rd, the keywords appear in the title of the articles, in the abstract or in the metadata, 4th, the keywords appear in the title of the articles, in the abstract or in their metadata, and 5th, the search protocol for the different databases analyzed used the same keywords organized in search strings with the Boolean operator “and.” These keywords were “digital transformation and SME’s and Services,” “digital transformation and kibs,” “digital technology and SME’s and services,” “digital technology and Kibs,” “open Innovation and SME’s and services,” “open Innovation and Kibs,” “transformational leadership and SME’s and services “and “transformational leadership and Kibs.

Three exclusion rules were applied to limit the content of the articles and documents. These were (1) Articles that are not research papers or literature reviews are discarded, (2). The selected manuscripts must have a direct relationship with the subject matter of the study, and (3) The selected articles must be clearly explained with the methodology proposing adequate ways of addressing the research topic and answering the research questions.

The results of the literature review attempt to limit the multidisciplinary nature of digital transformation to the operational processes and business models which Kibs companies use to achieve digitization. These are discussed below.

### The DT process in Kibs companies

The concept of DT due to technological innovation poses important challenges for business organizations and researchers in terms of the identification and management of digital business models because business activity is interrupted as a radical renewal of technology takes place (Porter and Heppelmann, 2014; Pang et al., 2019; Warner and Wäger, 2019).

Digital transformation is a process that involves the adoption of transformational digital technologies which effect the functions, skills and strategies of the organization (Lucas et al., 2013). Digital technologies are adopted, such as business management software, new collaborative digital platforms, big data, cloud computing and hyperconnectivity, which lead to digitalization and changes in organizational processes and functions (Burke, 2011). This type of innovation at an organizational level is considered a way to generate competitive advantages for companies (Liao et al., 2007; Colino et al., 2014; Le and Lei, 2019). Open design in digital technologies creates new ways of collaborating and interacting in the ecosystems in which the companies operate (Hanelt et al., 2021). Kibs companies adapt better to organizational and flexible structures that favour continuous change and adaptability as companies and their competitors increasingly rely on outsourcing to external entities (Vial, 2019).

Strategies must be adopted for leadership and knowledge sharing so that companies can increase their capacity for innovation (Ritala et al., 2018; Le and Lei, 2019). However, to accelerate internal innovations Kibs companies must rely not only on their internal knowledge base, but also on external knowledge about the OI approach (Chesbrough et al., 2006; Martinez-Conesa et al., 2017), in which environmental factors, such as the characteristics of the country, the sector and the consumers are taken into account (Hanelt et al., 2021). This is due to the fact that customers have access to a large amount of information about the services available for the development of information technologies (Prahalad and Ramaswamy, 2004). Business relationships have also been affected by the emergence of social networks which provide a more complex interaction with customers for businesses as they must interact with many customers at the same time (Guo et al., 2020) taking into account that the customer is an important part of the final results of innovation (Belkahla and Triki, 2011; Saura et al., 2020).

Pergelova et al. (2019) and Lopez-Nicolas et al. (2020) detected that there is only a small number of empirical studies dealing with the effect of gender on the technologies which are implemented in SMEs. Furthermore, existing research on the use of digital technology by women is largely ignored (Dy et al., 2017), and the few studies found conclude that gender is not a driving factor of innovation in the Kibs companies studied (Mas-Tur and Ribeiro Soriano, 2014), nor is the gender of managers considered a relevant factor for the performance of the digitization of Kibs companies (Ribeiro-Navarrete et al., 2021).

### Kibs business in the administrative management sector

Kibs companies offer highly qualified services that provide added value for customers, companies and individuals which means that they must have advanced technologies and innovative strategies in their organizations (Miles, 2005; Miozzo and Grimshaw, 2005). These services require expert knowledge, since they complement the production processes of a company and provide solutions to society while solving complex problems for clients (Ashok, 2018).

The Kibs business sector studied in this research is administrative management in Spain composed of R&D companies and administrative agencies that provide multidisciplinary professional services, although their main activity is providing tax and administrative services to satisfy government regulations.

R&D companies participate independently in innovative activities (Tseng et al., 2011) and provide the expertise and knowledge for developing technological innovation, fulfilling the three basic functions of Kibs innovation systems, firstly as facilitators of innovation when supporting companies in their innovation processes (implementing tax, administrative, accounting management software, etc.), then secondly as knowledge providers when transferring existing knowledge between the companies in the administrative management ecosystem (continuous training in the technological innovations applied) and thirdly as generators of innovation by playing a decisive role in the initiation and development of the services provided by the companies (implementing digital platforms that connect Public Administration Agencies with companies and allowing procedures to be used effectively and safely; Gallouj, 2002; He and Wong, 2009).

Administrative Management Agencies provide many standardized services, although the Kibs sector is considered difficult to standardise as solutions must be personalized and customer-oriented (Bettencourt et al., 2002). The reality is that highly customized professional service packages can be provided with productization (Salmi et al., 2008). Standard blocks of professional services are used to provide a service with special characteristics. In the literature, different models for the process of service production have been reported in which the following phases have been identified, first review the strategic objectives of the customer to design the services required, second, evaluate the needs of the clients, the markets and competencies of the organization to create the service product and its modular structure and third, assemble the service package (composed of different modules) as the content of the product, fourth, pricing and marketing plans for implementation of the service and, explain how the product is to be put into practice, and finally the fifth stage, monitoring and development of the services using different analyses, such as profitability-costs (Vaattovaara, 1999; Länkinen et al., 2006).

Kibs companies can provide highly personalized services, such as legal or commercial reports, or productised models, such as vehicle registration and tax presentation. Kibs companies can reuse existing knowledge and manage customer relationships to develop new services to obtain a competitive advantage (Salmi et al., 2008).

### OI and implications of technology with special reference to digital platforms

One of the most important topics in the literature on innovation is OI, because companies can use it to access external sources to add value and obtain profits (Dahlander and Gann, 2010).

Different approaches are used in the literature to understand this concept (Chesbrough, 2003). Some authors consider that external knowledge and internal R&D are the most relevant factors of OI. Other authors consider OI as the use of resources that are part of the company (Tidd, 2014) and cannot be imitated by competitors to increase competitive advantage (Barney, 1991). There are studies that highlight the cooperation between the different contributors from four areas of OI, companies, individuals, private entities and public institutions. (Chesbrough, 2003; Tidd, 2014; Bengtsson et al., 2015; Greco et al., 2016; Hossain and Anees-ur-Rehman, 2016).

OI management in Kibs companies should focus on a knowledge-based vision, offering organizations strategies to achieve a competitive advantage by using professional workers to achieve optimal organizational results (Singh et al., 2021).

OI combines inbound and outbound innovation (Popa et al., 2017) to help Kibs companies meet customer needs and outperform market competition. Incoming OI initiates exploratory learning in order to discover and exploit the technical knowledge of external sources, such as consultants, public administration and professional organizations (Cheng and Shiu, 2015; Popa et al., 2017). Outgoing OI can be used to exploit the knowledge generated within the organization with licensing, patents or contractual agreements (Lichtenthaler, 2009; Hung and Chou, 2013) to improve organizational performance. OI therefore requires highly qualified and skilled human resources and human capacities to cooperate, accept external sources of knowledge and offer their own knowledge for use by Kibs (Benešová et al., 2020).

The advent of digital technologies has considerably changed how organizations work (Wiesböck and Hess, 2020). Recent technological advances generate and manage massive amounts of data that were not available before (Loebbecke and Picot, 2015; Björkdahl, 2020) and organizations can now incorporate them into their business models using OI to manage all the relevant information for decision making (Dahlander et al., 2021). There are some organizations that already use this type of strategy, such as platform companies like Amazon, Google and Facebook (Cusumano et al., 2019) and also, some companies in the industrial sector (Sjödin et al., 2020), but other companies are still at an early stage and have to face important challenges. Among the most relevant challenge is adequate data management for the different services and needs, such as, the creation, capture and exchange of data in the company and with others (Björkdahl, 2020).

Open service innovation is increasingly based on data-driven business models (Dahlander et al., 2021). These are becoming increasingly important and OI is ubiquitous when interacting in an ecosystem in which different players cooperate (Leten et al., 2013; Chesbrough et al., 2014; Holgersson et al., 2018). Currently, OI does not just solve a particular problem with exterior help but requires new styles of organization for it to be implemented (Giannopoulou et al., 2011; Chesbrough, 2019).

One of the key organizational developments that transforms organizations in the way they capture and create value are digital platforms (Gawer and Cusumano, 2002; Hagiu and Wright, 2015; Parker et al., 2016; Cusumano et al., 2019). These are technologies developed by R&D companies which serve as the basis for other companies to create more complementary innovations (Gawer and Cusumano, 2014, p: 420). Transactions between participating companies create network effects by connecting previously unconnected groups (Gawer, 2014) and enabling R&D companies, who are the platform owners, to establish an effective innovative division of labour and provide standardized interfaces (software development kits) as well as intermediation mechanisms that bring together different users that support innovation to create value together (Adner, 2017).

### Knowledge management: Transformational leadership

Leaders are currently considered an important asset of companies for their direct relationship with the performance of the organization (Aragón-Correa et al., 2007). The literature describes different types of leadership, each of which has its own virtues and weaknesses, but transformational leadership brings many positive aspects to the company due to its contribution to innovation, organizational learning and the creative capacity of employees (de Jong and Den Hartog, 2007).

Transformational leadership was introduced by Burns in the 1980s (Jia et al., 2018). It is currently considered the most effective leadership style (Phong et al., 2018) because it affects the key elements of a company such as, knowledge management, human capital (Birasnav et al., 2011) and management and innovation performance (Nguyen et al., 2017; Jia et al., 2018).

Transformational leaders must use knowledge-based strategies to establish processes for the exchange of knowledge and experience between workers in Kibs companies. This allows the workers to acquire new skills and knowledge to achieve their objectives at a personal and organizational level (Liao et al., 2007; Lin, 2008).

The support of the leader is essential for a favourable climate of knowledge exchange among the employees of a company (Lin and Lee, 2004). The leader must attend to the intrinsic needs of workers and must therefore earn their trust and establish a model of conduct with collective goals which must prevail over individual ones (Armandi et al., 2003; Vera and Crossan, 2004). This type of leadership can develop and maintain a system of control that values and rewards creativity and innovation with appropriate performance measures and reward systems (Jung, 2001). This is more effective in disruptive environments as workers are able to cope with rapid changes in an uncertain environment (Nguyen et al., 2017). It has been shown in scientific studies that transformational leadership positively influences creativity and innovation (Khalili, 2016). These types of leaders can support innovation in companies by increasing the motivation and ability of the members to be creative and innovative (Jia et al., 2018), which is a very relevant feature for promoting organizational development in Kibs companies.

### Negative factors of technological innovation

The use of technologies is a fundamental part of DT implementation. It is the basis of the digitization of many different social contexts and institutions (Wilkesmann and Wilkesmann, 2018). It generates competitive advantages for organizations (Yu et al., 2017), greater labor flexibility and more autonomy for workers (Symon and Pritchard, 2015). However, company managers may use technology to track workers’ performance and behavior, which can cause problems when abusive control destroys motivation and work engagement (Sewell et al., 2012) as workers consider that monitoring affects their privacy.

Another growing concern in scientific literature is the social and ethical impact of the digital technologies (Winfield and Jirotka, 2018) which are being integrated into business organizations and society in general. Privacy violation is an area that has been studied on many occasions (Stahl and Wright, 2018) as many digital services rely on the data collected by technological tools to detect consumer behavior and using these tools can cause privacy infringements.

The implementation of technological innovation in public administration is crucial for the modernization of the public sector to meet the needs of the private sector and the general population (Lyudmila and Anzhela, 2022). Adopting technology is considered a challenge for the public sectors of many countries. The problem encountered by public administrations is that the implementation of new technologies requires institutional agreements to be approved by policy makers and this can delay the adoption process (Conradie and Choenni, 2014; Savoldelli et al., 2014). Other studies into digital administration have found obstacles for the implementation of technological tools due to a lack of confidence in technological devices (Twizeyimana and Andersson, 2019), the digital immaturity of the management of public institutions which, in turn, generates a greater workload and work stress (Kuhlmann and Heuberger, 2021), and the lack of security in the privacy and ownership of citizens’ data (Schwester, 2009; Mergel et al., 2016). All of these require an adequate legal framework that clearly regulates the access and use of data and defines accountability.

## Materials and methods

### Research design

The objective of this research is to find the conceptual bases on which DT is founded in a study of a professional group of Kibs companies. To do this, 18 different experts form different areas of the sector were interviewed. A qualitative content analysis methodology was chosen as it is frequently applied in research and includes the transcription of verbal data from interviews (Schreier, 2012).

The success of this research technique is based on its ability to limit the data extracted in the interviews to the concepts that describe the research phenomena. A conceptual model or system (Hsieh and Shannon, 2005) of the type of questions used is created which is then used to validate the reliability of this scientific technique.

The conceptual structure used in this research, which is considered essential for the success of a qualitative study (Cepeda and Martin, 2005), is shown in Table 1.

**Figure 1:**
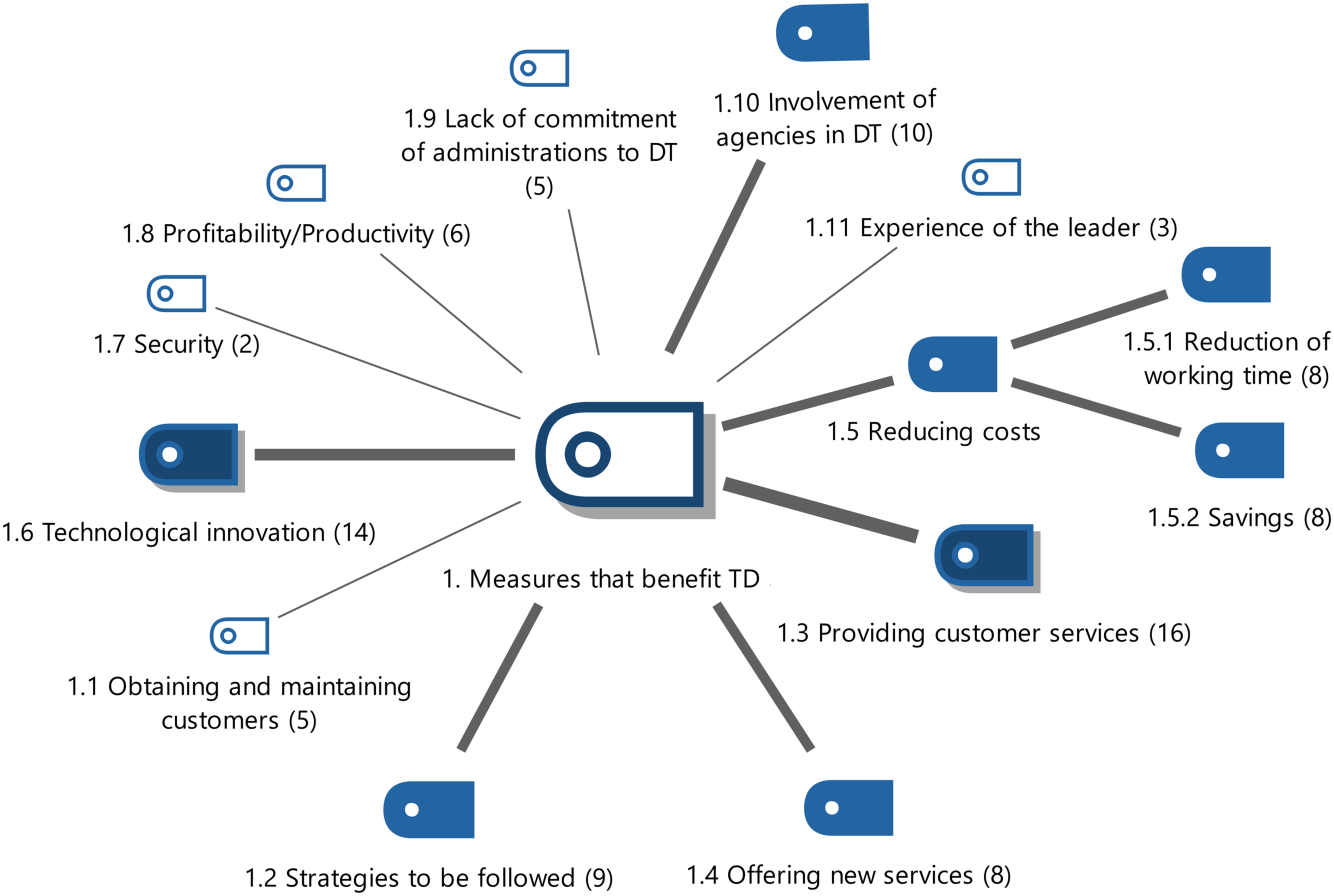
Measures that benefit DT with the weightings of the properties. Source: Authors own.

**Table 1:** Methodological phases of the research process.

1.Planification:	Qualitative Analysis: (Grounded Theory).
2.Data collection:	Research participants. Data collection.
3.Data analysis:	Data Coding.
4.Discussion and conclusions:	Considerations. Critical Analysis.

Source: Authors own.

The research method uses a 4-stage repetitive process to find the components of the DT structure in administrative management companies, which is one particular area of the conceptual framework (Miles and Huberman, 1994). The company environment will be investigated in order to obtain first-hand knowledge and allow the researcher to answer the research questions.

### Planning

A qualitative analysis using the grounded theory approach was used to collect and order the data obtained in interviews with the experts in a systematic way, building a formal theory based on social research (Glaser and Strauss, 2009). In this type of study, the researcher visualizes the data using an interpretation of social reality and reconstructs the experiences and meanings of the experts interviewed (Charmaz, 2006).

This methodology was selected because it is considered effective when studying a novel field of research in detail. In this study case, researchers do not have any advance knowledge of hypotheses that could answer the research questions for the subject and are therefore constructing a conceptual framework based only on the research data (Glaser and Holton, 2004). Likewise, the theory found with the analysis of DT of Kibs companies of other professional sectors, considered a macroenvironment, has not been analysed before in the scientific literature.

### Data collection

#### Research participants

The participants in this research were selected using intentional sampling, which is the most commonly used method for qualitative analysis (Elo et al., 2014). The interviewees were considered the most appropriate candidates to answer the research questions because they have the required expertise and knowledge about the subject being investigated.

The participants were professionals with extensive experience in the sector with different managerial functions in the professional associations of their region. Expert consultants, specialists in developing and implementing projects in the sector, were also included. This selection was broadened with the incorporation of a government official who works on managerial tasks in the special delegation of the tax office (AEAT) in Andalusia, Ceuta and Melilla and has been responsible for several technological projects for the digital transformation of the public administration. The varied nature of the elements that stimulate digital transformation has been taken into account along with the need to have data with the maximum level of traceability in qualitative research (Corbin and Strauss, 2014). Table 2 shows the details of the participants in the interview.

**Figure 2:**
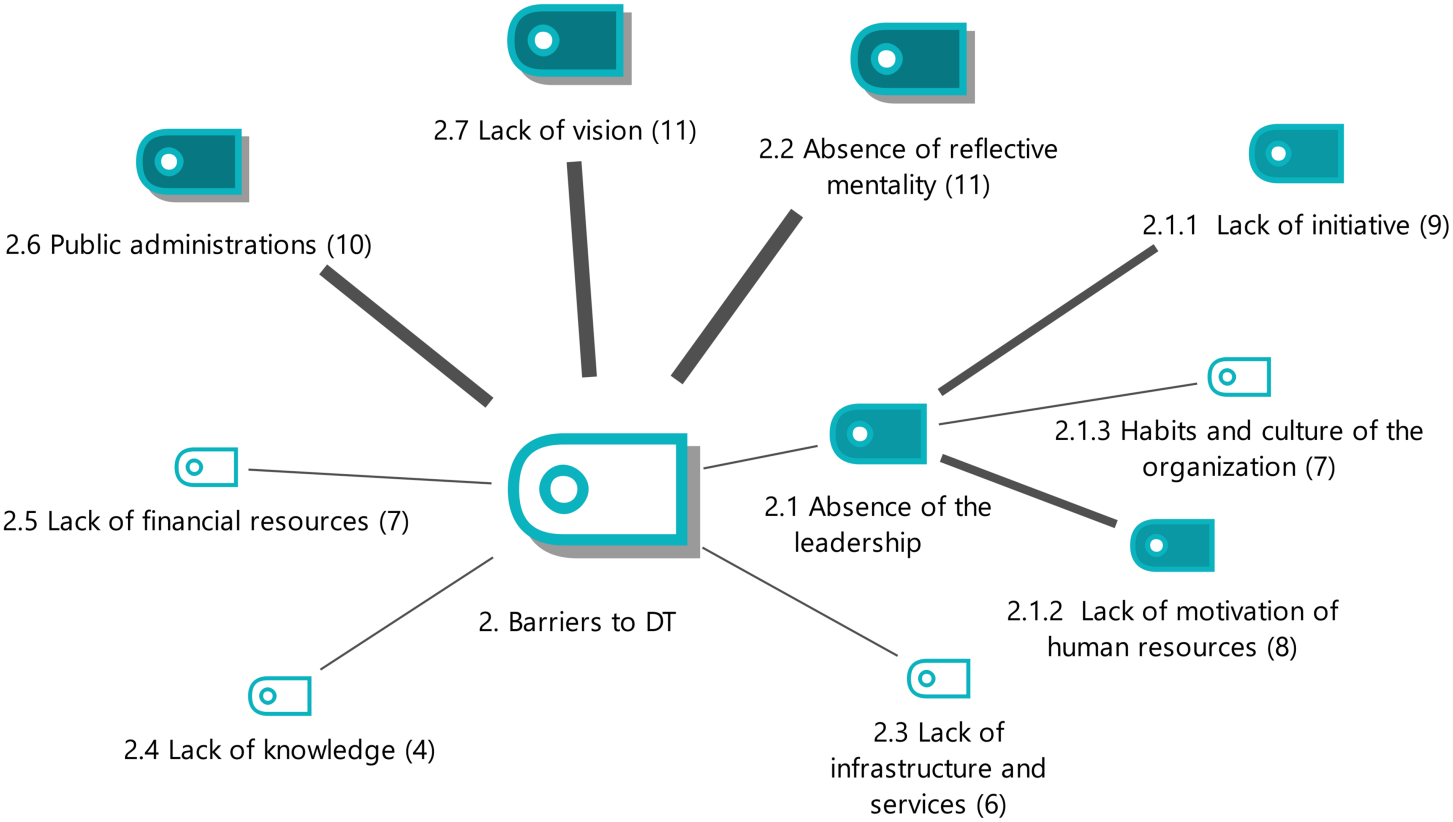
Barriers to DT with the weightings of the properties. Source: authors own.

**Table 2:** Profiles of the participants.

Participant	Interviewee
E1	Technical manager of a technological company in the services sector being studied.
E2	Manager of a technological company in the services sector being studied.
E3	Manager of a technological company in the services sector being studied.
E4	Project Director of the General Council for the service sector being studied.
E5	Professional with more than 20 years’ experience. Responsible for a professional association and the projects and procedures of the General Council.
E6	Professional with more than 25 years’ experience. Head of the territorial professional association and president of the General Council.
E7	Professional with more than 15 years’ experience. Head of the territorial Professional Association and the executive committee of the General Council.
E8	Technical manager of a technological company in the services sector being studied.
E9	Professional with more than 25 years’ experience. Head of the territorial professional association and secretary of the general council.
E10	Manager of a technological company in the services sector being studied.
E11	Manager of a technological company in the services sector being studied.
E12	Director of the General Council of the professional sector.
E13	Professional with more than 25 years’ experience. Responsible for a territorial professional association and the executive committee of the general council.
E14	External consultant on procedures and quality of professional associations.
E15	Professional with more than 30 years’ experience. Head of the territorial professional association and the executive committee of the general council.
E16	Professional with more than 15 years’ experience. Member of the Governing Board of the territorial professional association.
E17	Professional with more than 15 years’ experience. Member of the Governing Board of the territorial professional association.
E18	Affiliated Member. Specialist in electronic tax administration. Special Delegation of the tax office (A.E.A.T.) in Andalusia, Ceuta and Melilla.

Source: Authors own.

#### Interview development

Candidates were interviewed individually for between 40 and 60 min from June to September 2020 using a semi-structured questionnaire with open-ended questions ordered by complexity. These types of questions were chosen in order to facilitate the correct transcription of the interview data and a valid analysis following the rules of grounded theory (Glaser, 2002).

The collection of semi-structured data was chosen as the best research method in this case as it avoids bias in the interviews because the transcription is considered objective and is written without introducing any prejudices and respecting the privileges associated with using the information (Warr and Pyett, 1999).

The interviews were online using a video conferencing software application which recorded the interview after the interviewees gave their consent. Additional notes were taken in order to fully understand the interview and answers in context (Table 3).

**Figure 3:**
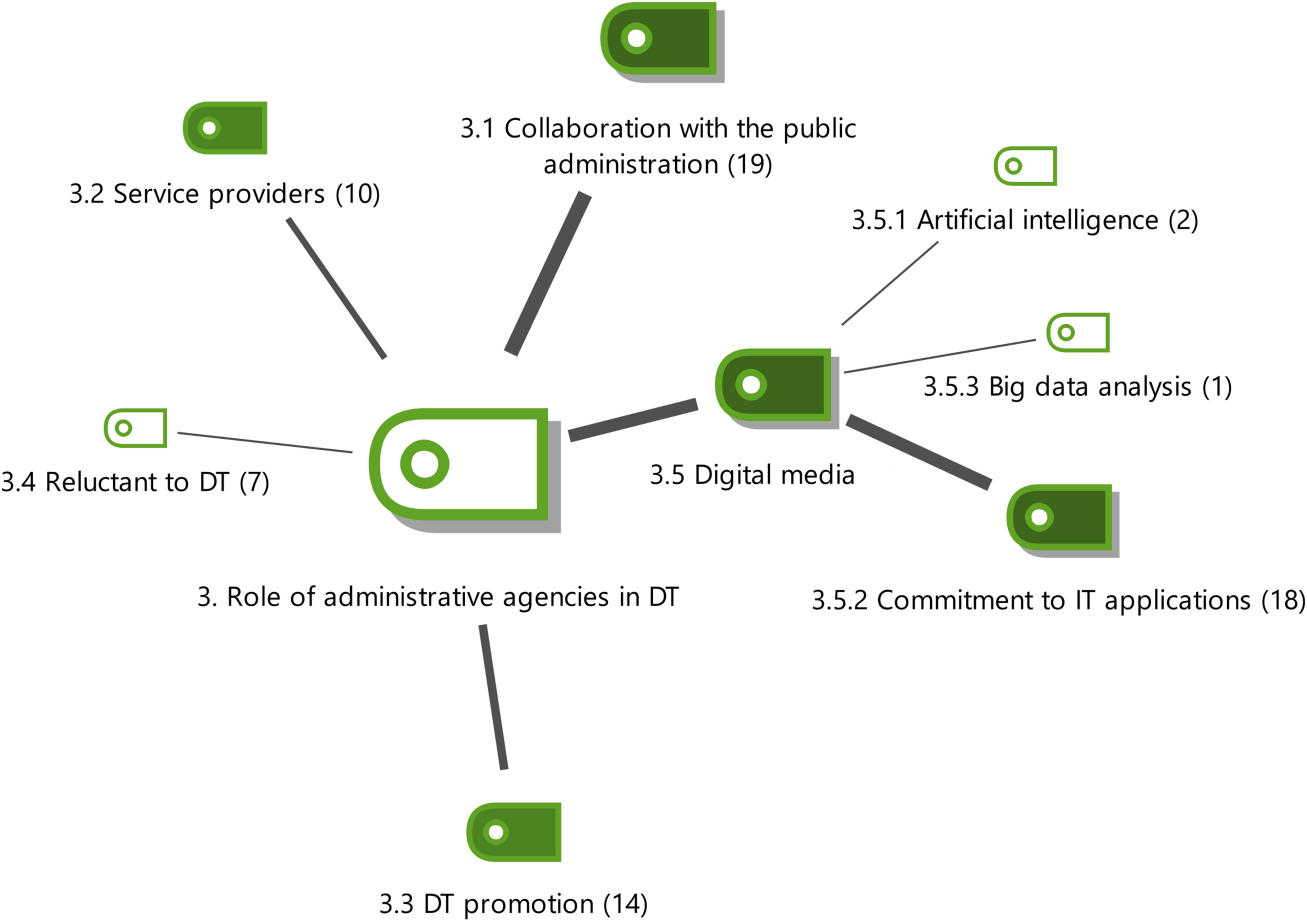
Role of the Administrative Agencies in DT with the weightings of the properties. Source: authors own.

**Table 3:** Interview guide (initial version).

Starting Questions		From author
a. What is Digital Transformation for you?	b. How does transformation differentiate from innovation?	Sousa-Zomer et al., 2020
**Questions**		
c. What are the main factors that motivate Kibs companies to seek this transformation?	d. What do Kibs companies want to achieve with this type of change?	Ko et al., 2021
e. What were the main steps taken in this transformation process?	f. What elements were a priority in your digital transformation process?	Vey et al., 2017
g. How important do you think it is to apply a digital maturity model to implement this type of transformation?	h. How would you define digital maturity and what kind of models do you know?	Minonne et al., 2018; Muñoz and Avila, 2019
i. Do you think that a company should follow an existing model or create its own that suits its needs? What does this decision depend on?	j. Did your agency decide to use a model of digital maturity or create one?	North et al., 2019; Dressler and Paunovic, 2020
k. What variables were used most in the transformation process?	l. Were there well-defined needs and/or objectives for transformation? What were they?	Ferreira et al., 2019
m. What were the main steps in the transformation process?	n. Which actions gave expected results and which ones did not? Why?	Greenwood et al., 2005
o. How involved in the process were/are the management/property/managers of the consultancy?	p. Based on your company’s digital transformation process, could you ensure that it has been successfully implemented?	Jung et al., 2008; Nguyen et al., 2017; Phong et al., 2018
q. How would you define success in the digital transformation of your agency?	r. Do you think that there are Spanish companies that have been successful in their digital transformation process? Which ones? Can you give one or two examples and explain why?	Loonam et al., 2018; Vial, 2019
s. Do you think that the professional administrative management sector has to adapt a lot to this type of change?	t. How do you think an administrative agency can succeed by using digital transformation?	Diller et al., 2020
u. Do you consider that the following variables have had the most impact on the success of the digital transformation in your company? • Technology • Organization • Client • Strategy • Culture • Operations • People • Capabilities • Innovation	v. What obstacles and/or resistance frequently arise in this process? w. How have you been able to face these types of obstacles?	Birasnav et al., 2011; Wiesböck and Hess, 2020
x. What lessons were learnt in the Digital Transformation process?	y. What achievements do you think were accomplished? z. What other achievements have not yet been accomplished and why?	Kronblad, 2019
aa. What comes next? What are the next steps after the first stage of the process? When does it end?	bb. Finally, how do you see the future of digital transformation in Spain? What do you think is necessary?	Frey and Osborne, 2017
Final question		
Are there any other comments you would like to make?		Closing question.

Source: Author’s own.

The initial or opening questions were used to analyse the conceptual approach to DT by the interviewees. The following questions were used to investigate which components of DT have the most impact in this professional sector.

Once the interviews had been transcribed, the answers given by the participants were summarized in order to further analyse the data and identify the ideas and concepts until theoretical saturation is reached when nothing new can be identified in the data (Glaser and Strauss, 2009).

### Data analysis

The data gathered from the answers given by interviewees was analysed using MAXQDA 10 software, as it is a powerful computer program for qualitative data analysis (Palos-Sanchez et al., 2022).

First, the transcripts and notes of the interviews were analysed sentence by sentence to identify the most outstanding experiences of the participants and understand the most important concepts of the subject (Glaser, 1998).

Second, the key segments of the data were then selected to extract and encode the most important words, sentences and paragraphs. After identifying the key points of the context (memos) by separating DT into discrete concepts, codes are assigned to the results. The continuous analysis of similar data from the interviews made it necessary to partially modify the initial codes and create a new coding system. Firstly, grouping codes because they are linked to each other, as in “promotion” and “advisor” to become “DT promotion,” as both terms express an intention to promote and advise on technological innovation. “Profitability” and “productivity,” “profitability/productivity,” “obtaining customers” and “maintaining customers” are unified into the code “obtaining and maintaining customers” as interviewees used these terms interchangeably to define the positive benefits for KIBS companies after the implementation of the technological processes of DT. New codes were created for terms that are connected although interviewees cited them independently. These new codes are: “reducing costs,” “absence of leadership,” “digital media” and “collaboration with government agencies.” Table 4 shows the initial coding and the subsequent modifications that condense the information in the analytical notes (memos) after the interviews and reorganizes the concepts.

**Figure 4:**
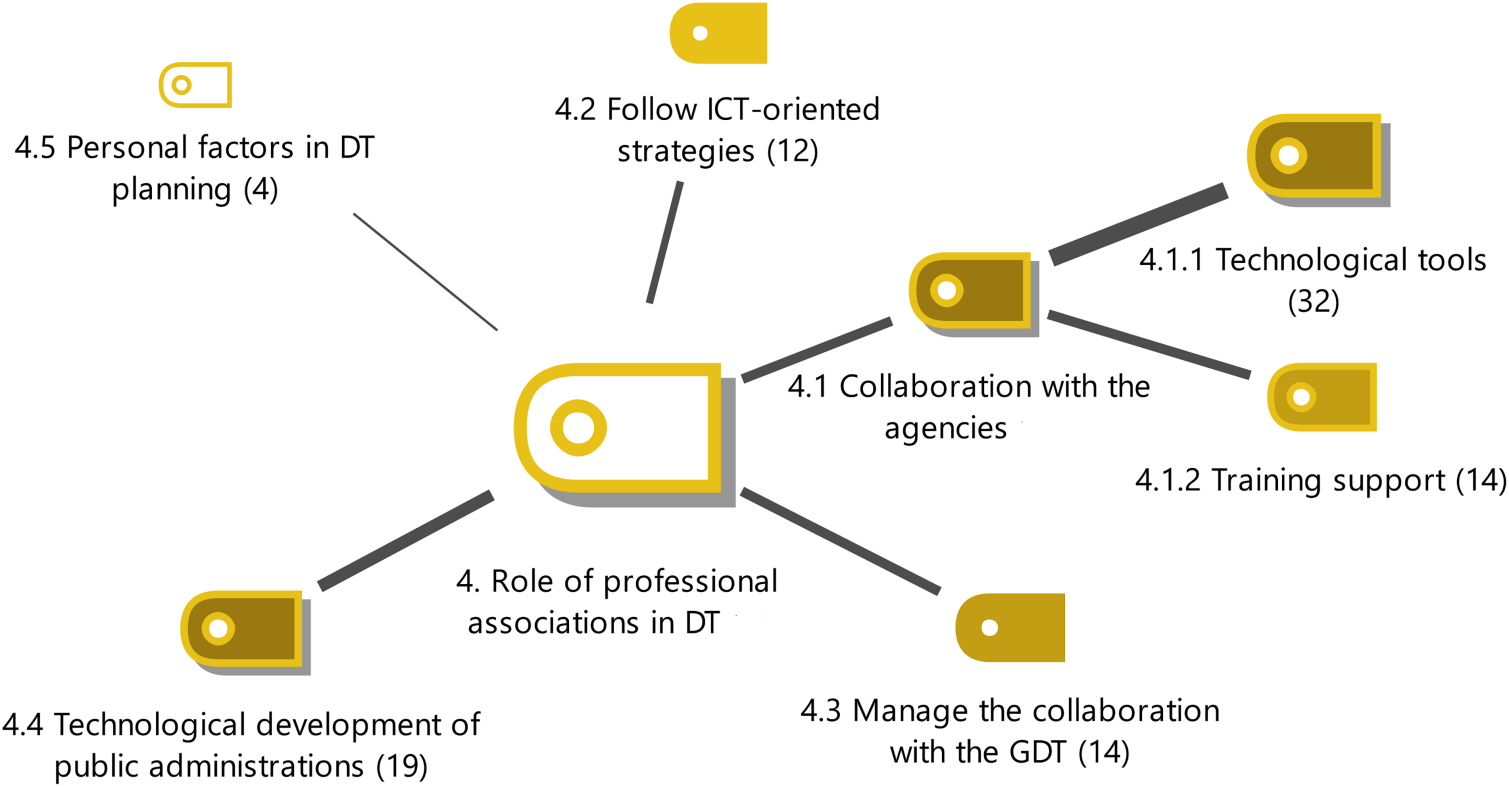
Role of professional associations in DT with the weighting of the properties. Source: Authors own.

**Table 4:** New codes developed from the initial codes.

Initial code	New code	Memo (short description)
Promotion.	DT promotion.	The Manager aligns the business for technological innovation processes and transmits this idea to all agents.
Advisor.		
Profitability.	Profitability/productivity.	The use of DT enablers generates higher productivity for organizations leading to increased profitability.
Productivity.		
Obtaining customers.	Obtaining and maintaining customers.	DT will help gain new clients, increase the loyalty of existing ones and improve the image of the agencies.
Maintaining customers.		
Reduction of working time.	Reducing cost: Reduction of working time. Savings.	Technological tools eliminate bureaucracy, red tape and staff travel. Digitizing means reducing business costs by improving management procedures, making them more efficient and competitive with online services.
Savings.		
Lack of initiative.	Absence of the leadership: Lack of initiative. Lack of motivation of human resources. Habits and culture of the organization.	The agencies are forced to innovate by the market and Public Administration. It is difficult for them to leave their comfort zone. The lack of motivation of workers who do not understand that using technological tools is necessary for the continuity of the agencies. Digitalization is hindered by maintaining traditional production processes.
Lack of motivation of human resources.		
Habits and culture of the organization.		
Artificial intelligence.	Digital media: Artificial intelligence. Commitment to IT applications. Big data Analysis.	Managers must implement digital artificial intelligence tools. Invest in and implement business management software, e.g., document management. The analysis of large amounts of data by any electronic device optimizes business processes.
Commitment to IT applications.		
Big data Analysis.		
Technological tools of the professional associations.	Collaboration with the agencies: Technological tools. Training support.	Professional associations invest in the technological development of useful software for the profession. Professional Associations provide technical training in DT for their members.
Training Support of the professional associations.		

Source: Author’s own.

The third step was selective coding. The constant comparison process identifies the complexity and diversity of the data (Glaser and Strauss, 2009, pp: 102–113). After completing the final conceptual coding, a further analysis of the interview notes (memos) reorganized and identified the categories and the concepts and the relationships between them (see Appendix 1). No new elements could be identified from the interview notes after the data analysis and so the theoretical saturation was considered valid(Glaser, 2009). The objective was to reach this level of conceptual analysis by discovering the central categories that organize the remaining subcategories in order to determine the formal theory (Lindsey, 2002).

Finally, the main categories were correlated with the results of a literature review to enrich the content of the categories detected in the inductive analysis.

### Quality of the grounded theory

To ensure the quality of the research data, the evaluation criteria were based on credibility, transferability, dependability and confirmability as established by Guba and Lincoln (1985; see Table 5).

**Figure 5:**
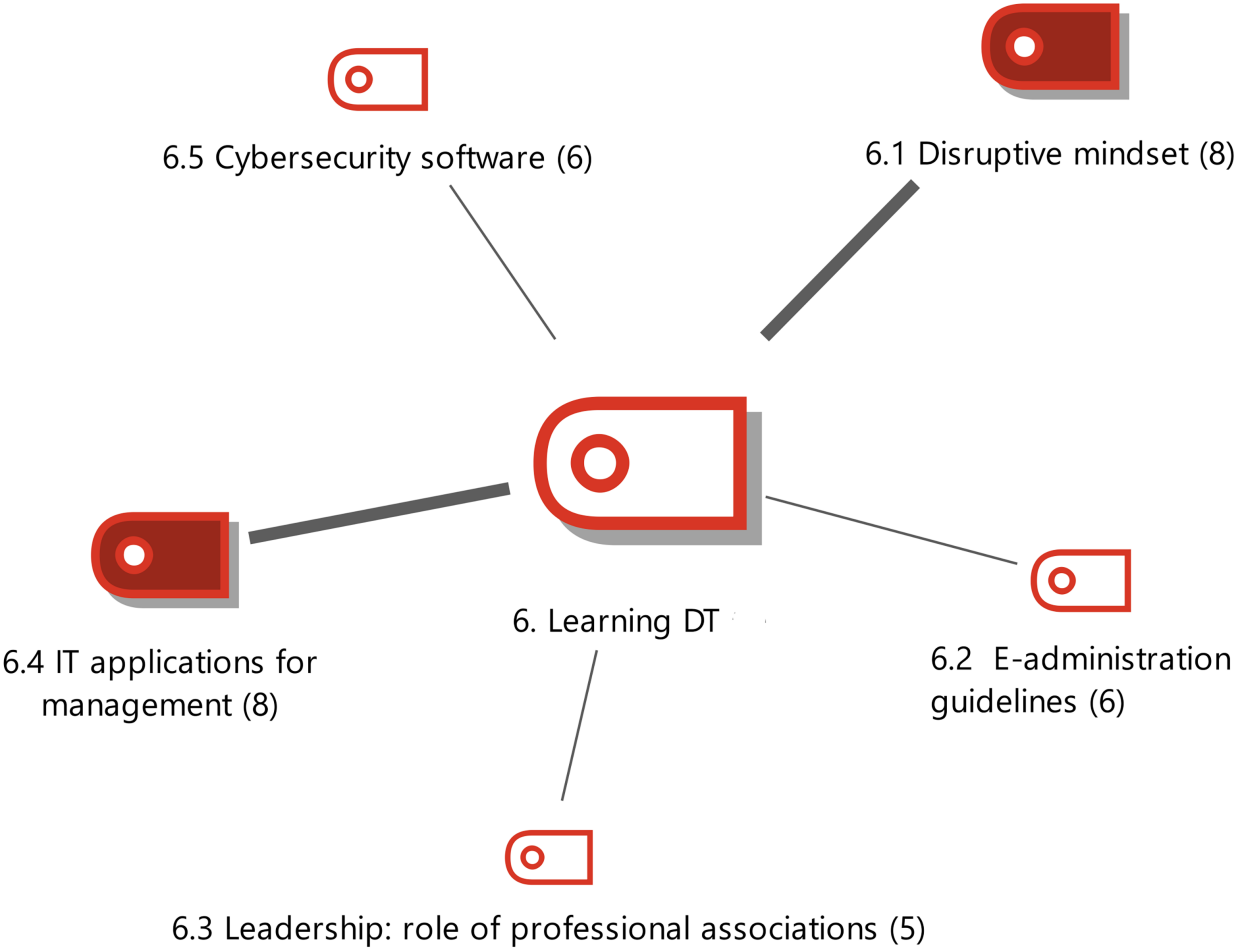
DT Learning with the weighting of the properties. Source: Authors own.

**Table 5:** Parameters used to ensure the quality of the grounded theory research.

Criterion	
Credibility	Open-ended questions were asked to interviewees about general aspects of the subject and then other questions about particular aspects of their profession so that the interviewee gave a variety of answers. The non-verbal behaviour of the participants during the interview was also transcribed. The transcription of the interview was shown to the interviewee to verify that the findings were correctly reported and to validate the information given before proceeding with the data analysis.
Transferability	A wide range of experiences were reported since managers and expert personnel from different Spanish DT companies were interviewed, as well as a public official with a managerial DT position in the public administration who could give a complementary vision of the subject to experts from private corporations. As a result, the in-depth analysis is considered suitable for the research.
Dependability	A detailed literature review was made of the study topic in order to adapt the questions of the qualitative analysis to the needs detected in the scientific literature. The study plan and the analysis methods were established by two researchers with extensive research experience. In addition, several reviews were made to ensure the results were consistent.
Confirmability	The written interpretation of the results of the study were shown to the interviewed expert for verification.

Source: Authors own.

This study uses the results obtained in interviews, that is, from the interaction between the researcher and the interviewees. The results of this causality must be rigorously elaborated, and the research method must use the guidelines shown in the table above to guarantee the effectiveness and efficacy of this research (Cepeda and Martin, 2005).

## Results

The data gathered from the interviews was used to identify the basic categorical framework of the subject. 2 basic concepts, 7 main categories, 43 first-level subcategories and 10 s-level subcategories were identified.

The main categories were: the measures that benefit DT, the barriers to DT, the role of administrative agencies in DT, the role of professional associations in DT, the level of digital maturity, DT Learning and the future of DT. These properties will be used to explain the most relevant aspects of the conceptualization of the factors that determine DT in the service sector in the study and the approach of this professional group to the new digital environment (see Table 6).

**Figure 6:**
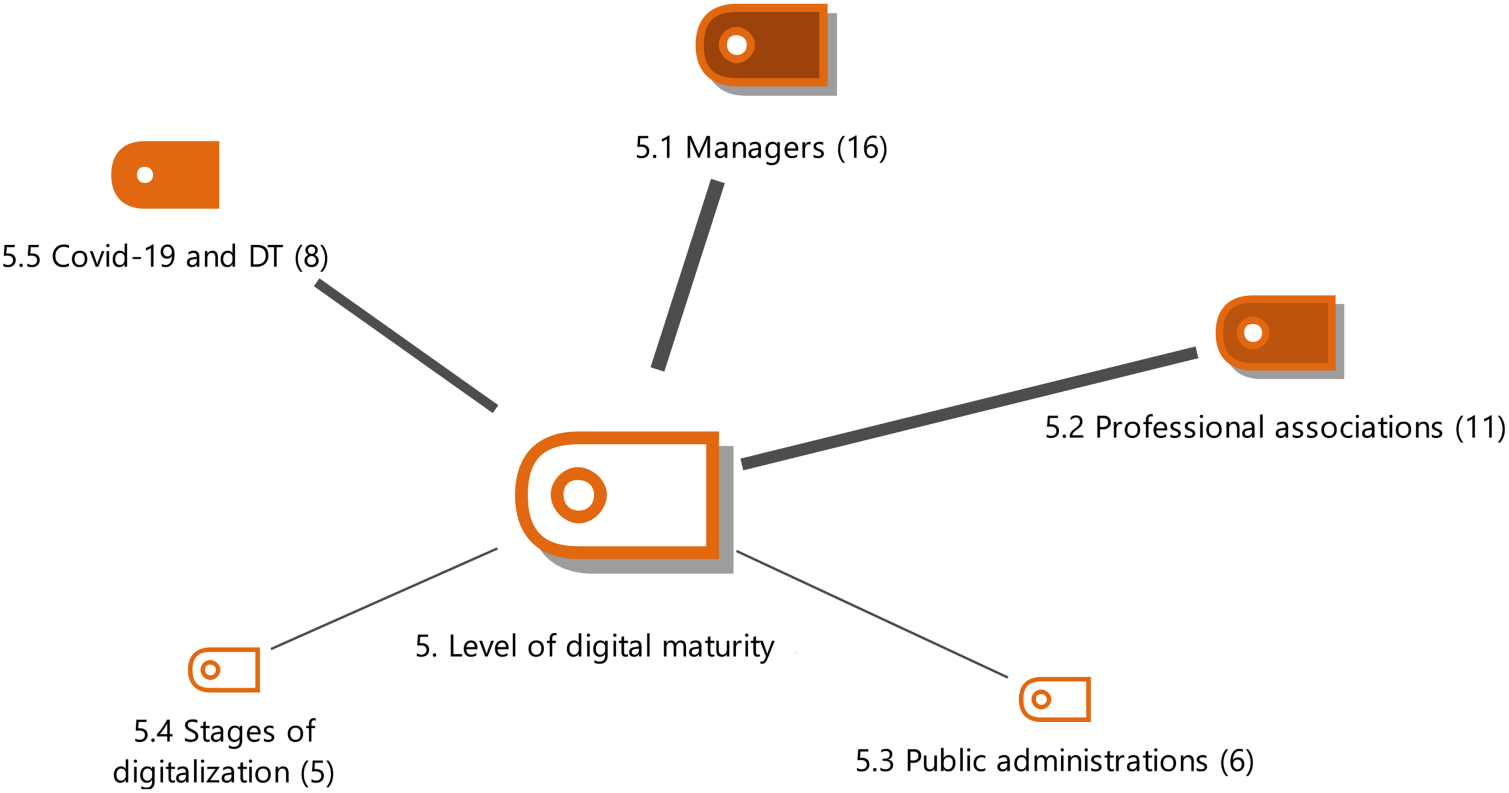
Level of Digital Maturity with the weightings of the properties. Source: Authors own.

**Table 6:** Conceptual research model.

Factors of DT collected in the qualitative analysis.				
Measures that benefit DT.	Barriers to DT.	Role of administrative agencies in DT.	Role of professional associations in DT.	Learning DT.
**Factors that will determine DT in the new digital environment.**				
Level of digital maturity.	The future of DT.			

Source: Authors own.

The results are presented using the code matrices (see Appendix 1) to design the conceptual structure. These were used to organise the main categories, subcategories and concepts discovered by categorization (Lindsey, 2002). In the analysis process and with densification (Strauss, 1987; Glaser and Strauss, 2009) subcategories arise due to constant comparison, which seeks continuous validation. These are irrelevant when explaining the phenomenon studied because they do not have a significant relationship with the main category. Although their conceptual relationships are represented graphically, the results are not commented on because they are not relevant when describing the main category.

### Factors of DT in categories

This first main conceptualization is found after a content analysis of the interviews with the experts. 7 different categories have been used to explain the concept.

#### Measures that benefit DT

11 subcategories were identified from the answers given by the experts interviewed. To conceptualize the category and study it, the original sample is reduced to 6 subcategories, considering the rest of the properties irrelevant.

The subcategories that are considered most important and that favour DT are: technological innovation and facilitating customer service (see Figure 1).

Technological innovation is necessary in order to succeed in this profession (E2, E3). Technology must be an integral part of the business solutions (E4). Technological innovation must be used to improve the solutions provided in an increasingly changing environment (E18). Innovation is necessary because it allows the client to interact efficiently with the public administration (E9, E11) in order to satisfactorily execute all the necessary governmental procedures (E1). DT is considered essential to facilitate customer service, and make the interaction between manager and client easier (E5, E16). The service is improved by automating processes (E14). Customers feel closer to the business (E3) and collaborative work and internal communication are encouraged (E13). In short, the use of technologies increases the quality of service and the speed of response with the client (E17).

Another property that was identified on many occasions was the involvement of administrative agencies with DT. This is based on the need to implement psychological elements of leadership to all members of organizations such as: motivation, conviction and need for change (E9, E13). The public administration is recognised as a necessary figure just as administrative agencies are considered necessary for DT (E6, E10). The Spanish General Directorate of Traffic (GDT) stands out as the public administration most involved in DT (E3).

The strategies to be followed must be oriented towards technological innovation (E7) with the implementation of management software to differentiate agencies from competitors (E2). The strategy must be based on the following elements: digital awareness, vectorization of the business and implementation of digital tools (E18).

Another outstanding aspect of code segmentation is the offer of new services that are similar to the one being developed, since customers must be offered innovative services with technological components (E8) due to the needs of the profession (E10) and thus clients’ needs are satisfied with the tax/administrative services provided (E14). This digital approach reduces costs incurred by organizations and improves competitiveness. This first-level subcategory is further divided into 2 second-level subcategories, which are:

- The reduction in working time optimizes and reduces the cost of personnel employed in the organization (E5, E12), and also allows reconciliation of family life (E2).
- The savings generated by the improvement in processing administrative procedures (E1, E2) also mean there is a cost reduction in economic terms (E5, E12, E16, E18). There is less bureaucracy in administrative procedures such as vehicle registration, which means that taxes are collected earlier and the fees paid can be recovered more quickly (E8).

#### Barriers to DT

7 subcategories are identified that define the barriers to DT. Only the most relevant criteria will be shown, for the 4 subcategories.

This section deals with the negative aspects of the economic environment that prevent the implementation of a coherent DT. The most significant aspect is that participants are concerned about the absence of a reflective mentality, which causes members to resist leaving their comfort zone (E3), so a change of mentality is required by introducing new ways of working and technological investment (E1). This situation occurs because the average age of administrative managers is high and they have a traditional view of organizations (E14). Therefore, a move must be made from a reactive mentality, when decisions are made once events happen, to a proactive mentality that takes the initiatives and is in alignment with technological processes (E16) (see Figure 2).

Another of the barriers to DT is a lack of vision which means that production processes are not changed to digital ones (E1). Companies do not feel the need to invest in technology (E4) nor innovate because they are not sure that there will be added value for customers (E8) and because they have a rigid mentality and an aversion to change (E6).

The experts pointed out that there are certain public administrations that are reluctant to incorporate DT (for example, the Civil Registry; E4), because they do not have enough technology to face the change (E10) and because their processes are very bureaucratic (E12).

Especially important for barriers to DT are 2 second-level subcategories (lack of initiative and lack of motivation of human resources) which are caused by the absence of leadership. This is shown by the inability of the administrative manager to motivate members of the organization, which leads to a lack of initiatives for innovation in the sector (E4, E1). The years of previous experience of senior professionals in this sector mean that they do not lead DT and are unable to convey its importance (E8) as it is difficult for them to leave their comfort zone (E12). There are organizations that have difficulty entering the world of eGovernment (E18). This absence of leadership means that the human resources of the agency lack motivation (E2), their acceptance of traditional methods (E10) and their reluctance to see digital tools as essential for their business (E16).

#### The role of administrative agencies in DT

5 main subcategories were identified in the theoretical sampling, with 4 essential subcategories for the explanation of the properties of the main category (see Figure 3).

The role of administrative agencies in DT is mainly due to their close relationship with the public administration (E7) since they have become facilitators of administrative relations between individuals and public administrations with a history of collaboration due to the agreements signed (E15). These existing alliances are imposed by law so this sector is privileged because there are an increasing number of procedures which can only be managed using electronic public administration (E18). The close collaboration with the GDT is an example of a paradigm of a public entity that has incorporated digitalization (E10).

Another very relevant role of administrative agencies in DT is the commitment to computer applications. This 2^nd^ level subcategory refers to the importance that experts give to the need to invest in technological tools such as cloud-computing, mobility, social media, document management, etc. (E18) and also emphasises the need to implement administrative management software (E15) like MobileGest, a mobile digital identity solution that allows the clients of consultancies to sign documents with full legal validity using a smartphone (E11, E13).

Professionals in this sector are providers of innovative services (E16) with technological components to satisfy the demands of customers (E3), especially for tax/administrative consulting (E4). Examples of this new type of services with technological components are the digital certificate (E11), applications such as MobileGest (E15) and technological e-commerce tools that are used to market and purchase services aligned with the new needs of clients (E18).

Organizations have a business perspective which includes technological innovation processes to promote DT (E3, E4). They have to provide useful projects like digital platforms that can interact with several administrations at the same time (E7). New telematic communication channels with the customer have to be created. The idea is to implement a new concept of the office (E15). DT is promoted by encouraging management to reengineer tax and administrative processes, systems analysis and verifying the effectiveness of technological tools and whether new software must be implemented (E17).

#### The role of professional associations in DT

5 subcategories were found in the model which are factors that explain the studied category. 3 subcategories were seen to be the most relevant to the explanation of the conceptual phenomenon.

The most decisive aspect of the level of involvement of professional associations with DT was seen to be collaboration with consultancies due to their position in the market. Experts have identified 2 attributes that define this subcategory, technological tools and training support (see Figure 4).

Professional associations help consultancies to implement efficient technological processes and develop technological tools with their R&D companies. Seville uses the Milenium Digital Platform for telematic registration of consultancies (E8). Another R&D company, SIGA produces computer tools and procedures that provide improvements for professionals in the sector especially in procedures with public administration for traffic (E16). It provides technological tools that optimize the administrative and tax procedures that manage vehicles (E3, E4). OEgam is an example of technological development with more than 20 million euros funding and a staff of 80 professionals. The Professional Association of Madrid launched this telematic platform to streamline and optimize procedures in different areas. A digital project called E-Mandato has also been launched to implement digital transformation of key processes of agencies such as the representation mandate (E6). The technological tool MobileGest (E1) is also an important development. Experts also commented on the evolution of office management software for payroll, invoicing, accounting, tax filing, etc. (E2, E5) and the creation of a nationalities platform to streamline the procedures for granting Spanish nationality by residence (E4).

Professional associations also give training support to their members for the parts of DT that are considered most relevant (E4, E5). The training is for the new work procedures and protocols of advisors (E6) and the technological tools involved (E11, E18). Professionals in the sector can improve their efficiency after this training. There is a foundation in Catalonia that offers official master’s degrees for the profession (E7).

Another subcategory is the technological impulse of the public administration that professional associations collaborate with, promoting internet connectivity, eradicating paper and reducing administrative work. The driving force of the profession is the public administration, which greatly influences technological activity in the sector (E14). Public administration is generally slower in integrating technological adaptation and relies on the more flexible professional sector to implement digital processes (E11).

The most substantial collaboration with public administration is with the GDT (E5). This organisation has seen an improvement in the efficiency of its procedures with the introduction of DT (E11) and is considered to be at the forefront of DT in the profession. Efforts have been made to develop the digital platform and the document management system that are used to formalize the procedures of this administration (E16). The existing agreements with the GDT are historical and require all administrative procedures for circulation, registration, transfers and vehicles to be done with telematic procedures (E15).

There is consensus among the participants interviewed that professional associations should follow ICT-oriented strategies. A technological plan must be followed which is suitable for the needs of the public administration department (E1) with a framework agreement and guidelines so that digital identity is ensured (E11). The strategies must be adapted to new technologies and tasks to streamline administrative procedures for individuals and companies (E6). Strategies must be found that adapt new technologies to help professionals by implementing understandable processes for the advisor and creating added value (E7). The aim is to create a plan for software, management and information security systems that standardizes processes and promotes ICT projects (E14).

#### DT learning

The last category of the main concept was found to be linked to 5 subcategories with special attention paid to the 2 most conceptually relevant for the data analysis.

DT Learning is defined as the procedures and technological tools in which organizations in this sector have to be trained to adapt to DT. This category has a very strong relationship with 2 properties called disruptive mentality and computer applications for management (see Figure 5).

In order to understand DT, professionals in this sector must change their traditional idea of administrative processes to include digital optimization (E4). Bureaucratic procedures must be changed for processes that generate technological innovation and allow instantaneous management of administrative files (E13). A disruptive mentality allows digital change by adopting new habits that include small modifications of behaviour in order to adjust the workers’ mentality to the new paradigm. This should be done collaboratively as teamwork is important in the process (E18). Technological innovation will be accepted once the benefits of DT have been experienced (E1).

Computer applications for management must be learnt in order to fully understand the implications of DT. It is essential that this type of software is learnt as the applications are an important source of information and therefore, very useful for advisors (E7, E10). Dexterity in the use of computer equipment (desktop scanner, laptops) is another necessary requirement (E8). A series of platforms must be incorporated into the company so that professionals can interact with clients and in turn communicate the information to the public administration service (E13).

### Factors of DT in the new digital environment

Another important consideration is the direction that DT should take in the future. In this analysis, 2 fundamental categories have been established for the actions of the interviewees and contribute to the formulation of the substantive theory.

#### Level of digital maturity

This is the degree of technological implementation existing in a profession, especially in administration agencies and professional associations. The effect of COVID on the level of maturity of organizations in this sector is studied (see Figure 6).

Most of the interviewed experts consider that the level of digital maturity is medium-low (E1, E4, E7, E8). It is also evident that, although the level of digitalization is not optimal, this maturity is considered more advanced than for other professions such as lawyers, but less developed than in banking (E17). Experts generally consider that larger companies have the highest levels of digital maturity in this economic sector.

Professional associations show a high level of digital maturity compared to the sector studied and are the fundamental pillar of the digital development of administrative agencies (E4). These institutions are well positioned in digital maturity (E1) with a medium-high level (E6, E7, E17). Professional associations, followed by administrative agencies, have the highest level of digital maturity in the sector (E17).

Another aspect of DT is the influence of the Coronavirus on the factors of digitalization (COVID 19 and DT). COVID has been seen to have played an important role in the DT of agencies (E18). It has accelerated the digitalization process and has triggered the process of transformation of new technological mechanisms to connect agencies, public administration and customers (E17). COVID is a factor that has activated digitalization (E4, E8, E13).

#### The future of DT in the sector

**Figure 7:**
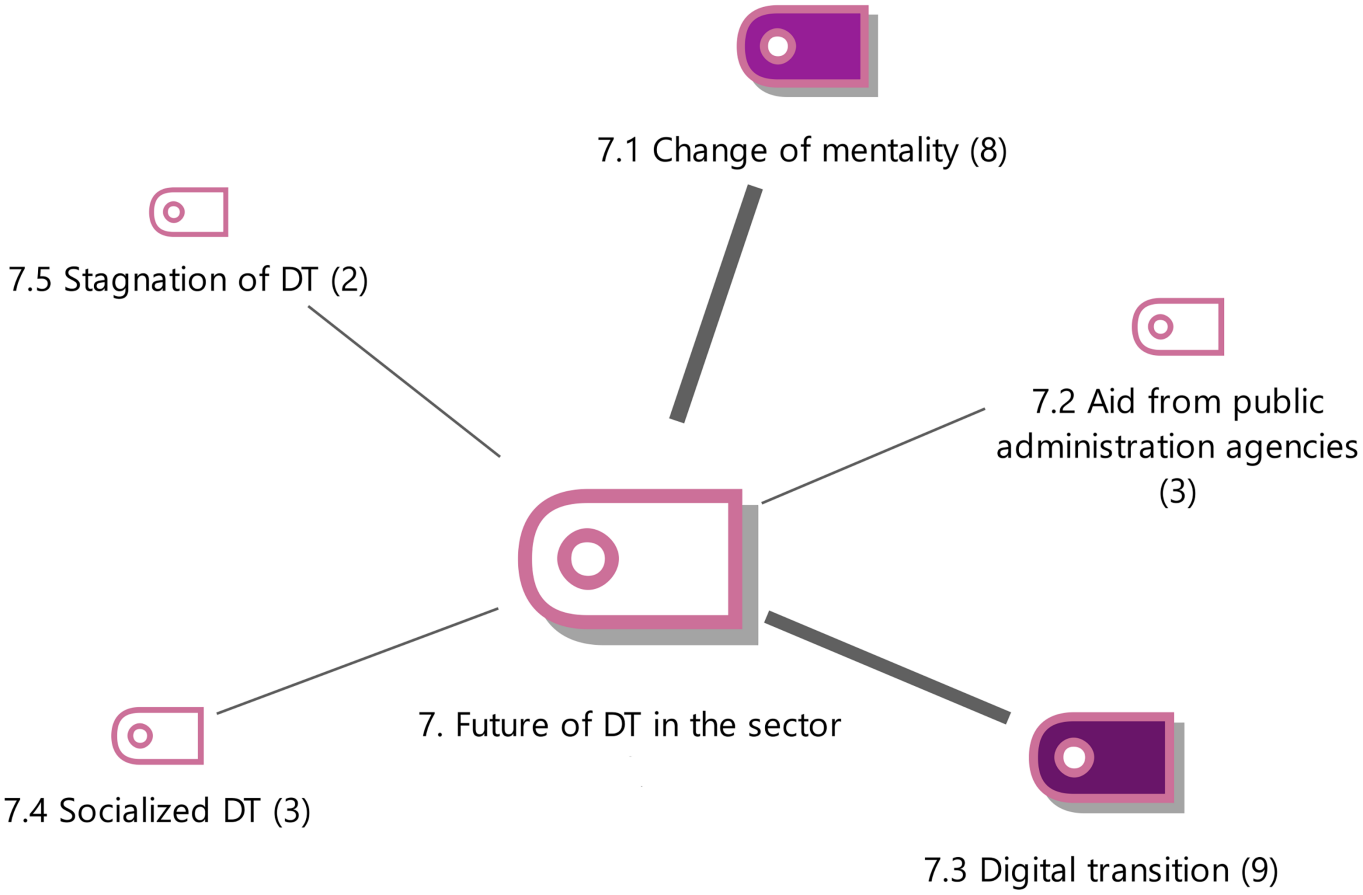
Future of DT in the sector with the weighting of the properties Source: Authors own.

The future of the administrative managers sector is believed to depend on 2 essential elements, digital transition and change of mentality (see Figure 7).

Digital transition is a current reality that professionals have to accept and use in order to generate high added value (E1). The sector is concerned with digitizing the administrative/tax procedures between the citizen and public administration agencies (E2). The future of this sector will include artificial intelligence as a new type of technological process (E7, E9) and big data will also be gradually incorporated (E9). These can improve the competitiveness of professional companies in the sector by enhancing digital processes in the organization and integrating ERP (Enterprise Resource Planning) with clients (E16).

A change of mentality is necessary so that professionals convince themselves that digitalization is the way to manage customers competitively and paper can be forgotten (E10). DT is an opportunity for change for advisors since this professional sector is based on legal aspects with little innovation (E4).

### Weighting of theoretical concepts

The aim of a grounded theory is to reach the third level of conceptual analysis. The first level is the collection of data, the second is the generation of categories and the third level is discovering the central category that organizes the rest of the categories, from which a higher level is reached, the formal theory.

7 categories were analysed and the most relevant, from the analysis of data using the segmentation process, were found to be the role of professional associations in DT and measures that benefit DT. The next most relevant were the role of administrative agencies in DT and Barriers to DT with similar weightings (see Appendix 2). These are the most representative categories of the analysis and they are all connected to one of the main theoretical concepts, factors which will determine DT in the new digital environment (see Appendix 3).

### Comparison of the main categories found and the literature review

When analysing the main categories of the qualitative data gathered in the interviews, it was observed that the results show coincidences with the theoretical concepts obtained from the literature review associated with the subject (see Table 7).

**Table 7:** Properties of DT from the qualitative analysis and associated with the scientific literature.

Measures benefiting DT (Kibs companies).	Authors
Commitment to technological innovation.	Hekkert et al., 2007; Bergek et al., 2008; Brynjolfsson and Mcfee, 2014
OI strategy that allows collaboration and creation of value for the professional sector.	Van De Vrande et al., 2009; Luo et al., 2017; Scuotto et al., 2017; Zhang et al., 2017; Singh et al., 2021
Digital platforms as a technological tool that transform organizations.	Cusumano et al., 2019; Dahlander et al., 2021
The client, as the central axis of the companies, uses digital technology as it improves the company-client interaction.	Corver and Elkhuizen, 2014; Muñoz and Avila, 2019
The behaviour of the leader due to psychological aspects.	Judge et al., 2002; Diller et al., 2020
A transformational leader, who positively influences the company workers and favours innovation and creativity.	Lin and Lee, 2004; Khalili, 2016; Phong et al., 2018
Close collaboration with public administration agencies.	OECD, 2016; Troshani et al., 2018
Digitalization is assigned an important role in the business strategy.	Feher et al., 2017; Vial, 2019
Barriers to DT (Kibs companies).	Authors
Absence of Transformational Leadership.	Bass, 1990; Judge and Bono, 2000
Resistance by the organization.	Vey et al., 2017; Von Leipzig et al., 2017; Hai, 2021
Working conditions of the employees.	Erol et al., 2016; Shamim et al., 2016
Technological deficiencies, mainly in data security and privacy.	Newell and Marabelli, 2015

Source: Authors own.

DT is a concept with multiple aspects and points of view (Sugahara et al., 2017) and is frequently used by both researchers and practitioners.

## Discussion

### Comparison with other studies

Two central questions about the present and future factors of DT in Kibs companies were studied in this research. In the literature there is research that analyzes the implementation of certain components of DT in law firms, but based on the cost–benefit effect. It is worth highlighting the study by Breunig and Skjølsvik (2017) who consider only innovation as the central axis of DT, originating improvements in communications and marketing of the services provided by organizations. Hongdao et al. (2019) analyze digital technologies to facilitate accessibility with their customers. Other studies consider a business opportunity the implementation of certain technological tools to reach more customers, thus we highlight Campbell (2012) that through software such as Legal Zoom optimizes the provision of legal services. Williams et al. (2015) and Brivot et al. (2014) highlight the business opportunity generated by creating web platforms to reach real virtual law firms such as Trademarkia, or to direct innovation to real technological systems for knowledge management such as KMS (Knowledge Management System), offering more standardized services.

Leadership style and personality have been studied in the literature in the context of digitalization as elements affecting the digital maturity of tax consultancies in Germany (Diller et al., 2020).

Innovation management through OI has been treated by researchers as a system of exchange and collaboration in the development of Kibs companies (Van De Vrande et al., 2009; Allahar, 2019).

In the literature we can highlight studies that through an inductive analysis explore the technological changes of Kibs firms. In this sense, Durczak et al. (2022) through an exploration based on grounded theory show the obstacles presented by lawyers towards digital innovation. Brooks et al. (2018), through the constant comparative method as a grounded theory approach, highlights the importance of implementing artificial intelligence in the legal services sector due to the needs to innovate due to the arrival of new data-driven technologies, detecting cultural and structural barriers that hinder its implementation. Leckel et al. (2020) study certain public initiatives of regional networks, which facilitate collaborative innovation through dissemination and promotion mechanisms to implement OI strategies among SMEs. In order to obtain a deep understanding of their research they understand that the most appropriate research method to analyze this reality is through grounded theory.

Previous studies have analysed certain elements of DT (organizational strategies, technological tools, psychological factors that determine the behaviour of the leader and OI) as inherent disruptive sources in organizations. In our case, the conceptual contribution that is made is not only about companies, but is based on a broader social context which is a complete professional sector. In addition, there are no known scientific contributions that analyze the three factors studied (technological tools, leadership and OI) as essential elements of the DT of Kibs companies.

## Conclusion

This study evaluates the critical factors that conceptually define DT for the professional sector of Kibs companies. The research uses the grounded theory methodological approach. The objective of this methodology is not to conceptually define DT, but to establish its most relevant components using substantive data collected from interviews and a literature review.

The results of the interviews underline the heterogeneity of the processes that make up DT. A double conceptual classification is made. First, all the elements that construct the factors of DT are grouped together and then, the level of digitization in the sector is defined (a non-relevant concept due to the low frequency of responses from the interviewees).

The factors needed to generate DT are the most interesting pieces of information in this analysis. Unlike previous studies, this research has been able to identify the most relevant of all the factors studied in order to define the concept of DT. The positive approach shown by the respondents when empirically defining DT should be noted. The category “measures that benefit DT” is explored in depth, using the empirical development of technological innovation that generates better customer interaction, customer loyalty and a reduction in organizational costs. Another important element of the inductive analysis is the “role of professional associations in DT.” In this category some new concepts are explored such as collaboration with the agencies, manage the collaboration with the GDT and technological development of public administrations. It is mainly concerned with the technological development and digital training of Kibs companies which are part of the professional sector and creating synergies and promoting e-Government with value networks. These are the indicators that generate DT at the organizational level in the macro-environment studied and are associated with the theoretical process of OI.

Other results show that the coded information for the components “digital media,” “IT applications for management and cybersecurity software,” which are then grouped into the categories of “role of administrative agencies in DT” and “learning DT” partially overlap, thus developing the same theoretical concept for digital technology which is a necessary factor for DT.

Finally, the theoretical contribution related to transformational leadership is broadly developed by several subcategories. First, there are those that reflect a positive conceptual view of DT. Three subcategories are selected (experience of the leader, disruptive mindset and leadership: role of professional associations) that provide overlapping ideas in the meaning developed by this concept, highlighting the benefits of properly managing DT through a leader who motivates and convinces his or her organization of the need to digitize. Secondly, and as an additional contribution to leadership, but with negative connotations in the conceptual delimitation of DT, other codes appear in this study called: absence of the leadership, absence of reflective mentality and lack of vision, which are shown in the category “barriers to DT,” and in its inductive development is associated with the difficulties that arise in the implementation of DT in an organization; if they do not follow strategies oriented towards transformational leadership.

### Theoretical implications

**Figure 8:**
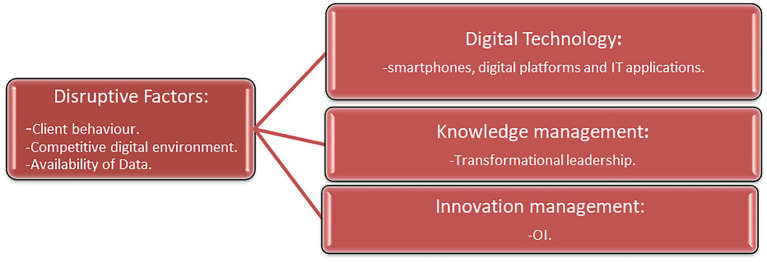
Factors needed for successful DT. Source: Authors own.

This study shows that the digital transformation changes in this professional sector are a combination of digital technology, transformational leadership and OI (see Figure 8). These factors are relevant for the new competitive environments of this service sector. One of these is the need to implement a transformational leadership style to generate a direct, positive effect on organizational innovation and provoke an increase in performance (Jung et al., 2008). With this leadership style, knowledge management is created (Lindsey, 2002) involving all members of the company in digital processes.

### Practical implications

The results of this research can be useful for managers of an SME or micro-SME which provides consulting services as a part of a professional group. The study highlights the transcendental aspects of DT development at the intra-organizational level. In line with the proposed conceptual development, the strategic and managerial requirements must foster the creation of networks connecting small-sized companies in the same professional sector. It is important to generate highly competitive technological infrastructures which would otherwise not be implemented due to the high financial and design costs which these companies could not support individually.

Policy makers can take advantage of the implications obtained in this study and assume a leadership role to promote the comprehensive development of e-Government. Strategic positioning by governments can be an opportunity to improve the flow of information and the with companies. The objective is to increase the agility and reduce the bureaucracy of the functions of some public administrations. In order to do this public funding is needed, along with a complete communications network with professional groups so that information about the innovation processes can be exchanged.

Finally, another suggestion is to use a big data technological enabler, because the results show that this technological tool should be implemented more effectively. Using this type of service will help to achieve a well-managed transition to DT since the sector has a large amount of data available and should be able to diagnose and integrate information using this tool.

### Limitations and future research

Once the general factors that influence DT in this professional group are analysed, the study could be expanded to investigate more factors that might limit or prevent OI, such as analysing the ethical and legal requirements of the transfer of data ownership that might be an obstacle to collaboration between Kibs companies. Another area that should be investigated is the possible abuse of power caused by the compulsory use of technological platforms because there are no other alternatives available for clients to carry out certain administrative procedures, such as vehicle registration plates. This is a very relevant topic because an increasing number of traditional industries, like the automotive one, are facing similar issues (Björkdahl, 2020).

Another interesting aspect for investigation is how Kibs companies that use R&D services invest internal resources. The qualitative organization of companies, the motivation and rewards for workers and the challenges for managers could be analysed regarding them as stimulants of innovation and identifying the possible, resulting effects generated in the sector.

The limitations of this research include the fact that the conclusions of the grounded theory analysis are based on data that comes from limited interviews with experts who were selected non-randomly so that it cannot be claimed that their conclusions are universal.

A similar limitation is that the literature analysed in the study has been selected by the authors of this work. This subjectivity can be seen as a limitation of the research because the systematization and impartiality of the analysis of scientific contents has been transgressed (Bigné, 1999).

## Data availability statement

The original contributions presented in the study are included in the article/Supplementary material; further inquiries can be directed to the corresponding author.

## Author contributions

All authors listed have made a substantial, direct, and intellectual contribution to the work and approved it for publication.

## Conflict of interest

The authors declare that the research was conducted in the absence of any commercial or financial relationships that could be construed as a potential conflict of interest.

## Publisher’s note

All claims expressed in this article are solely those of the authors and do not necessarily represent those of their affiliated organizations, or those of the publisher, the editors and the reviewers. Any product that may be evaluated in this article, or claim that may be made by its manufacturer, is not guaranteed or endorsed by the publisher.
