# Supplementary material for: A study of the factors which influence digital transformation in Kibs companies
Source: Front Psychol. 2022 Dec 20;13:993972. doi: 10.3389/fpsyg.2022.993972 (PMC9821610; doi:10.3389/fpsyg.2022.993972)
Supplement: Supplementary file 1 [file Data_Sheet_1.pdf]

Appendix 1. Matrix of Codes used in qualitative analysis of experts.

Code system	E1	E2	E3	E4	E5	E6	E7	E8	E9	E10	E11	E12	E13	E14	E15	E16	E17	E18	Sum
1. Measures that benefit DT																			0
1.1 Obtaining and maintaining customers	3				1									1					5
1.2 Estrategies to be followed		1				1	1				1			1	1		1	2	9
1.3 Providing customer services	2	1	3	1	1		1		1		1		1	1		1	1	1	16
1.4 Offering new services		1				1		1		1		1		1		1		1	8
1.5 Reducing costs																			0
1.5.1 Reduction of working time	2	1			1			2				1						1	8
1.5.2 Savings	1	1		1	1			1				1				1		1	8
1.6 Technological innovation	1	1		1		1	1	2	1		1	1	1		1		1	1	14
1.7 Security								1										1	2
1.8 Profitability/Productivity	1	2					1	1				1							6
1.9 Lack of commitment of administrations to DT				1			1		1		1				1				5
1.10 Involvement of agencies in DT			2			1			1	2			1		1		1	1	10
1.11 Experience of the leader						1			1	1									3
2. Barriers to DT																			0
2.1 Absence of the leadership																			0
2.1.1 Lack of initiative	2	1		1				1				1	1					2	9
2.1.2 Lack of motivation of human resources	2	1			2					1						1		1	8
2.1.3 Habits and culture of the organization		1			1	1		2									2		7
2.2 Absence of reflective mentality	3		1	1				1		2			1	1		1			11
2.3 Lack of infrastructure and services								1	1	1		2						1	6
2.4 Lack of knowledge	2														1		1		4
2.5 Lack of financial resources	1	1			2	1				1							1		7
2.6 Public administrations				2						2	1	1	2		1		1		10
2.7 Lack of vision	3			1		1		1	1					1		1		2	11
3. Role of administrative agencies in DT																			0
3.1 Collaboration with the public administration			2	1		1	1		1	2	3		2		2	1	2	1	19
3.2 Service providers		1	1	2						1	2				1	1		1	10
3.3 DT promotion	1	2	1	2	1		2		1				2		1		1		14
3.4 Reluctant to DT	2	1												2		1	1		7
3.5 Digital media																			0
3.5.1 Artificial intelligence		1				1													2
3.5.2 Commitment to IT applications		2	2	1	2	1			3	1	2		1		1	1		1	18
3.5.3 Big data analysis																		1	1

Code system	E1	E2	E3	E4	E5	E6	E7	E8	E9	E10	E11	E12	E13	E14	E15	E16	E17	E18	Sum
4. Role of professional associations in DT																			0
4.1 Collaboration with the agencies																			0
4.1.1 Technological tools	4	6	1	2	1	3	4	2	2	2	1	1	1	1		1			32
4.1.2 Training support		1		1	1	1	2	3		1	1				1	1		1	14
4.2 Follow ICT-oriented strategies	1	1		2	1	2	1				1			1		1	1		12
4.3 Manage the collaboration with the GDT		3	1	1	1	1		2			1	1			2	1			14
4.4 Technological development of public administrations	2	2		1	1	2				1	3	1	1	1	3		1		19
4.5 Personal factors in DT planning									4										4
5. Level of digital maturity																			0
5.1 Managers	2			1	1	1	2	2	1	2		1	1			1	1		16
5.2 Professional associations	2			1		1	1		1			1	1	1			2		11
5.3 Public administrations	1			1				2				1	1						6
5.4 Stages of digitalization		1	1			1			1									1	5
5.5 Covid-19 and DT	1	1	1	1				1					1				1	1	8
6. Learning DT																			0
6.1 Disruptive mindset	1		1	1	1						1		1				1	1	8
6.2 E-administration guidelines		1				1			1	1								2	6
6.3 Leadership: role of professional associations	1			1							2		1						5
6.4 IT applications for management			1				1	1		1			1		1	1	1		8
6.5 Cybersecurity software	1	1	1		1									1				1	6
7. Future of DT in the sector																			0
7.1 Change of mentality	2			2						2								2	8
7.2 Aid from public administration agencies		2	1																3
7.3 Digital transition	1	1	2			2	1		1							1			9
7.4 Socialized DT											2		1						3
7.5 Stagnation of DT												2							2
Σ SUMA	45	39	22	30	20	26	20	27	23	25	24	17	22	13	18	17	21	28	437

Source: Authors own .

Appendix 2. Relevance of the categories by segmentation of the analysed data.

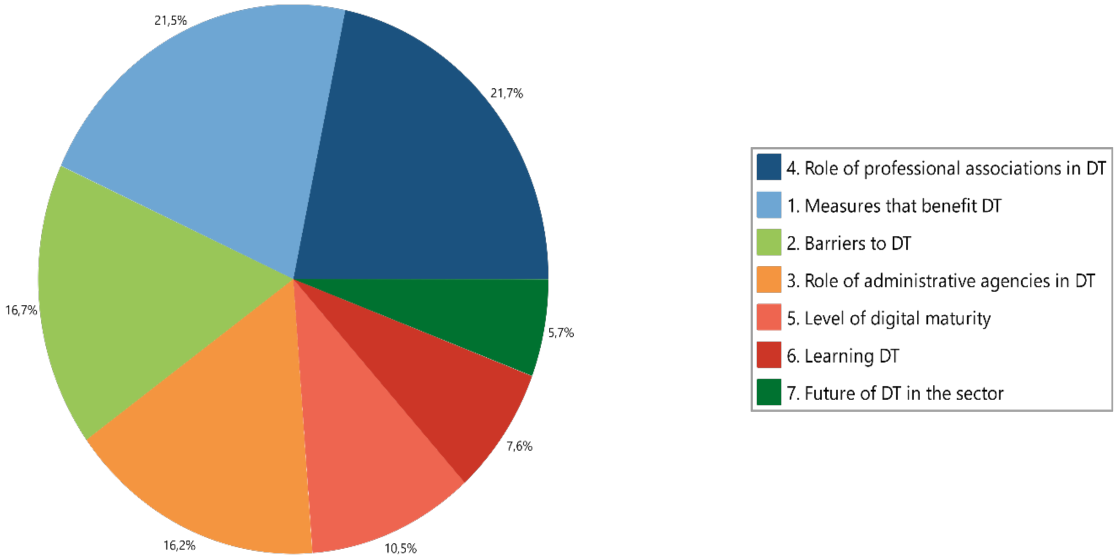

Source: Authors own.

Appendix 3. Word Cloud organised by the frequency of codes related to the Determinants of DT collected in the qualitative analysis.

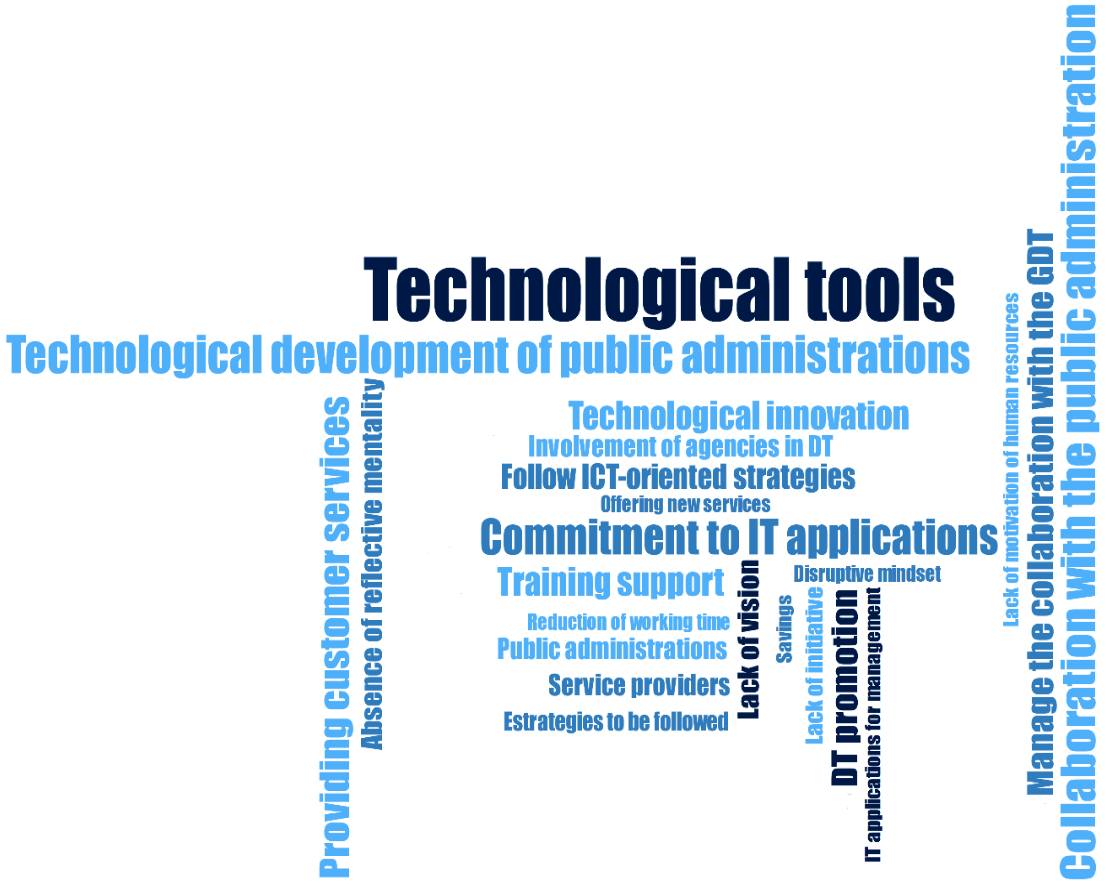

Source: Authors own.
